# Icaritin Synergistically Enhances the Radiosensitivity of 4T1 Breast Cancer Cells

**DOI:** 10.1371/journal.pone.0071347

**Published:** 2013-08-15

**Authors:** Jinsheng Hong, Zhenhuan Zhang, Wenlong Lv, Mei Zhang, Chun Chen, Shanmin Yang, Shan Li, Lurong Zhang, Deping Han, Weijian Zhang

**Affiliations:** 1 Department of Radiation Oncology, First Affiliated Hospital, Fujian Medical University, Fuzhou, Fujian, China; 2 Department of Radiation Oncology, UF Shands Cancer Center, Gainesville, Florida, United States of America; 3 Division of Radiation Biology, Central Research Lab, First Affiliated Hospital, Fujian Medical University, Fuzhou, China; 4 Department of Pharmacology, School of Pharmacy, Fujian Medical University, Fuzhou, Fujian, China; Faculty of Pharmacy, Ain Shams University, Egypt

## Abstract

Icaritin (ICT) is a hydrolytic form of icariin isolated from plants of the genus *Epimedium*. This study was to investigate the radiosensitization effect of icaritin and its possible underlying mechanism using murine 4T1 breast cancer cells. The combination of Icaritin at 3 µM or 6 µM with 6 or 8 Gy of ionizing radiation (IR) in the clonogenic assay yielded an ER (enhancement ratio) of 1.18 or 1.28, CI (combination index) of 0.38 or 0.19 and DRI (dose reducing index) of 2.51 or 5.07, respectively. These strongly suggest that Icaritin exerted a synergistic killing (?) effect with radiation on the tumor cells. This effect might relate with bioactivities of ICT: **1)** exert an anti-proliferative effect in a dose- and time-dependent manner, which is different from IR killing effect but likely work together with the IR effect; **2)** suppress the IR-induced activation of two survival paths, ERK1/2 and AKT; **3)** induce the G2/M blockage, enhancing IR killing effect; and **4)** synergize with IR to enhance cell apoptosis. In addition, ICT suppressed angiogenesis in chick embryo chorioallantoic membrane (CAM) assay. Taken together, ICT is a new radiosensitizer and can enhance anti-cancer effect of IR or other therapies.

## Introduction

Icaritin (ICT) is a hydrolytic form of icariin purified from the *genus Epimedium,* a traditional Chinese herbal medicine [1], [2], [3]. ICT has a broad range of biological activities, including the ability to stimulate neuronal and cardiac differentiation [4], [5], [6], enhance osteoblast differentiation, suppress osteoclast differentiation and activity [7], prevent steroid-associated osteonecrosis [8], induce apoptosis in human prostatic smooth muscle cells [9], and exert neuroprotective effects [10]. Moreover, it has also been reported that sub-micromolar concentrations of ICT exhibited estrogen-like activity in estrogen receptor-positive breast cancer MCF-7 cells [11] while it inhibited the growth of PC-3 prostate cancer cells [12]. Several studies supported that the ICT exerted an anti-proliferative effects in a variety of tumor cell lines, including human endometrial cancer cells [13] and HepG2 hepatocarcinoma cells [14], as well as chronic myeloid leukemia *in vivo* [15]. Recently, ICT was reported to induce cell death in activated hepatic stellate cells through mitochondrial-activated apoptosis, and also ameliorate the development of liver fibrosis in rats [16].

Ionizing radiation (IR) therapy is one of mainstream anti-cancer approaches for most types of malignancies, exerting the efficacy on local cancer control and reduces disease recurrence [17]. Extensive efforts have been undertaken by researchers to identify novel radiosensitizing and radioprotective drugs to enhance the therapeutical gain by reducing the radiation dose and side-effect and increasing the toleration of cancer patients to the treatment IR dose. For example, we have found that triptolide enhanced the anti-tumor effects of irradiation in pancreatic cancer *in vivo* and *in vitro* [18], [19]. It is unknown whether ICT exerts a synergistic effect with irradiation in any type of cancer. This study explored the ability and mechanism of ICT to enhance the therapeutic effects of IR using the malignant murine 4T1 breast cancer cell line.

## Materials and Methods

This study was approved by the relevant institutional review boards for animal study in Fujian Medical University. All experimental procedures were conducted in conformity with institutional guidelines for the care and use of laboratory animals in Fujian Medical University, Fuzhou, China, and conformed to the National Institutes of Health Guide for Care and Use of Laboratory Animals (Publication No. 85–23, revised 1985).

### Reagents and Cell Line

ICT (purity of 98.5%) was purchased from Shanghai Usea Biotech Company (China). A stock solution of 100 mM ICT was prepared in dimethyl sulfoxide (DMSO; Sigma, St. Louis, MO, USA) and stored at −20°C. The stock solution was diluted to the appropriate concentrations with culture medium. The final concentration of DMSO was less than 0.1% (vol/vol). The same amount of DMSO was used as the vehicle control throughout this study. The murine breast cancer cell line 4T1 (ATCC, Manassas, VA, USA) was maintained as monolayers in DMEM medium containing 10% heat-inactivated FBS with 1% penicillin/streptomycin at 37°C in a humidified atmosphere of 5% CO_2_.

### MTT Cell Proliferation Assay

MTT assay was performed as previously described to determine the survival of cultured cells [20]. The 4T1 cells were seeded into 96-well tissue culture plates at a density of 5–10×10^3^ cells per well, treated with ICT or vehicle for 24, 48 or 72 h, and then incubated with 50 µl of 0.5 mg/ml 3-(4,5-dimethylthiazol-2-yl)-2,5-diphenyl-tetrazolium bromile (MTT) solution for 4 h. The supernatant was discarded, and DMSO was added to each well. Absorbance at 570 nm was measured using a SpectraMax M2 reader (Molecular Devices, CA, USA). Each treatment and time point was triplicate and repeated twice times; the data shown is the representative of three independent experiments. The number of viable cells in the control group or at the initial time point was assigned a relative value of 100%.

### Clonogenic Survival Assay for Combination Effect of Icaritin and Radiation

Murine 4T1 cells were treated with vehicle alone, ICT alone (0, 1.5, 3, or 6 µmol/L), radiation alone (0, 2, 4, 6 or 8 Gy) or combination of ICT with radiation. The cells were plated in 60 mm dishes at different densities, based on the stringency of the treatments. The number of cells was adjusted to generate 50 to 200 colonies per dish at each radiation dose. After 14 days, the colonies were stained with crystal violet, and the numbers of colonies (containing ≥50 cells) were counted using Image-Pro® Plus. The surviving fraction (SF) was calculated as the ratio of the number of colonies to the number of cells plated (plating efficiency) in the treatment group, divided by the plating efficiency in the vehicle group. D_0_ (the incremental dose required for reducing the fraction of colonies to 37%, indicative of single-event killing, was calculated using the formula of the single hit multi target (SHMT) model [SF = 1 − (1 − e^−(D/D0)^)^n^ (20). SF_2_, the surviving fraction of exponentially growing cells when irradiated at the clinically relevant dose of 2 Gy; the enhancement ratio (ER); the combination index (CI) and dose reducing index (DRI) were calculated using CompuSyn (Biosoft, Cambridge, UK). Analysis of the synergistic effects of ICT with different doses of radiation was performed using Chou-Talalay’s combination index-isobologram and multiple drug-dose effect analysis, as described [21]. A CI <1 and DRI >1 indicated synergistic effects of ICT with IR.

### Cell-based ELISA-like Assays for Levels of Phosphorylation of ERK1/2 and AKT

ERK1/2 and AKT are involved in the regulation of proliferation [22]. To further investigate the mechanism of the radiosensitization effect of ICT on 4T1 cells, a cell based-ELISA-like assay [23], [24] was performed to quantify the levels of p-ERK1/2 and p-AKT [25]. The same numbers of 4T1 cells were cultured in triplicate in 96-well plates to 80% confluence; treated with vehicle alone or Icaritin at 3 or 13 uM for 4 or 24 hours, and then irradiated with 0, 1, 4, or 6 Gy. Ten minutes (for p-ERK1/2) or one hour (for p-AKT) later, the cells were fixed with 4% formalin for 5 min followed by 3% H_2_O_2_ quenching the endogenous peroxidase for 10 min and blocking with 1% of bovine albumin in 0.1% Tween-20-phosphate buffer for 15 min. After wash 3 times, the rabbit antibodies specifically against phosphorylated ERK1/2 or p-AKT (0.5 µg/ml) were added to the different wells overnight at 4°C followed by HRP-anti-rabbit IgG (0.2 µg/ml) for 1 hour at room temperature and then TMB substrate and stopper and read at A_450_ using a SpectraMax M2 reader.

### Cell Cycle Analysis

After treatment with ICT alone (0, 12.5, 25 µM), IR alone (0, 4, or 6 Gy), or ICT combined with IR for 72 hours, cells were harvested and immediately fixed in 70% alcohol overnight, then stained with PI (Propidium Iodide) solution contained 1% RNase A and 0.3% Triton-X100 for 30 min at room temperature. The samples were then measured by Accuri C6 flow cytometry (BD Biosciences, San Jose, CA) for the fractions of G_1_, S and G_2_M phase. The data were analyzed with C6 software. Data was representative of two independent experiments.

### Cell Apoptosis Analysis

4T1 cells were exposed to ICT alone (0, 12.5, 25 µM), IR alone (0, 4, or 6 Gy), or combination of ICT with IR for 72 hours, then harvested. After wash with cold PBS, cells were stained with annexin V (Biolegend, San Diego, CA) for 30 minutes and then with PI for 10 minutes according to the manufacture’s instruction. The staining samples then were subjected to Accuri C6 flow cytometry analysis.

### Chick Embryo Chorioallantoic Membrane Angiogenesis Assay

The effect of ICT on angiogenesis was investigated using the chick embryo chorioallantoic membrane (CAM) assay. The fertilized eggs were incubated at 37°C for eight days and then divided into groups (8 eggs/group), 100 µl of 0, 10, 20 or 40 µM ICT was added to the top of CAM. After incubation for 3 more days, the CAM of each alive egg was harvested and placed individually in 6 well plate. Images of the blood vessels of each CAM were captured using Motic Images Plus® (MediaCybernetics® Bethesda, MD, USA). The blood vessel area as a percentage of the total CAM area, blood vessel length and number of branch points were quantified using Image Pro® analysis software (MediaCybernetics® Bethesda, MD, USA).

### Statistical Analysis

All data are expressed as mean ± standard deviation (S.D.). The level of significance between two groups was assessed using Student’s *t*-test. The difference among three or more groups was assessed with one way ANOVA with SPSS 13 (Chicago, IL, USA). *P* values <0.05 were considered statistically significant.

## Results

### ICT Inhibits the Growth of 4T1 Breast Cancer Cells in a Dose- and Time-dependent Manner

**Figure 1:**
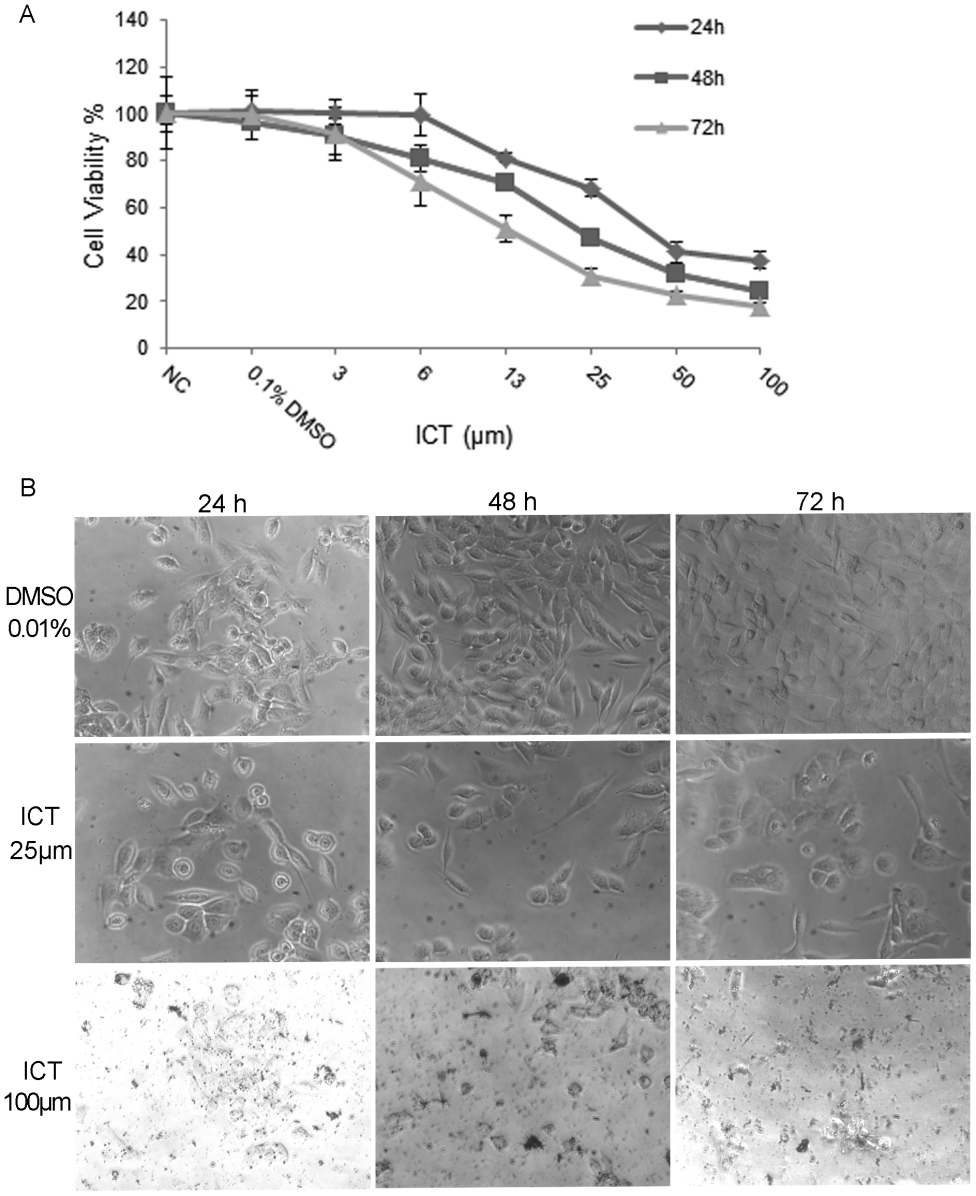
ICT inhibits the growth of 4T1 breast cancer cells in a dose- and time-dependent manner. The 4T1 cells were seeded into 96-well plates at a density of 5–10×10^3^ cells per well, treated with vehicle alone or ICT at the indicated different doses for 24, 48 or 72 h, and then the cell viability was determined by MTT assay. (A) ICT inhibits the growth of 4T1 breast cancer cells in a dose- and time-dependent manner. (B) Representative images of 4T1 cells treated with the indicated concentrations of ICT for 24 h, 48 h or 72 h.

To explore the interaction of ICT with IR, we have to know the effect of ICT alone on the testing 4T1 breast cancer cells, which will help to design the combination study with IR. The 4T1 cultured in triplicate in 96 well plates were treated with various concentrations of ICT for 24 h, 48 h or 72 hours, and then the survival cells were measured using the MTT assay. An ICT dose-dependent and time-dependent reduction of 4T1 cells was observed (Fig. 1). The concentration for 50% inhibition (IC_50_, mean ± SD) was 58±5 µM, 27±3 µM and 15±2 µM at 24 h, 48 h and 72 h, respectively. The data indicated that ICT inhibited the proliferation of 4T1 cells in a dose-dependent and time-dependent manner. To further explore if this effect is universal, we have also examined anti-proliferative effects of ICT on several other human cancer cell lines, including MCF-7 and MDA-MB-231 breast cancer cells, DU-145 and PC3 prostate cancer cells, as well as B16 murine melanoma cells. All results indicated that ICT suppressed the growth of cancer cells tested (data not shown), suggesting that ICT is likely to inhibit various types of cancer cells.

### ICT Acts Synergistically with Radiation on Suppression of Reproductive Growth of 4T1 Breast Cancer Cells

**Figure 2:**
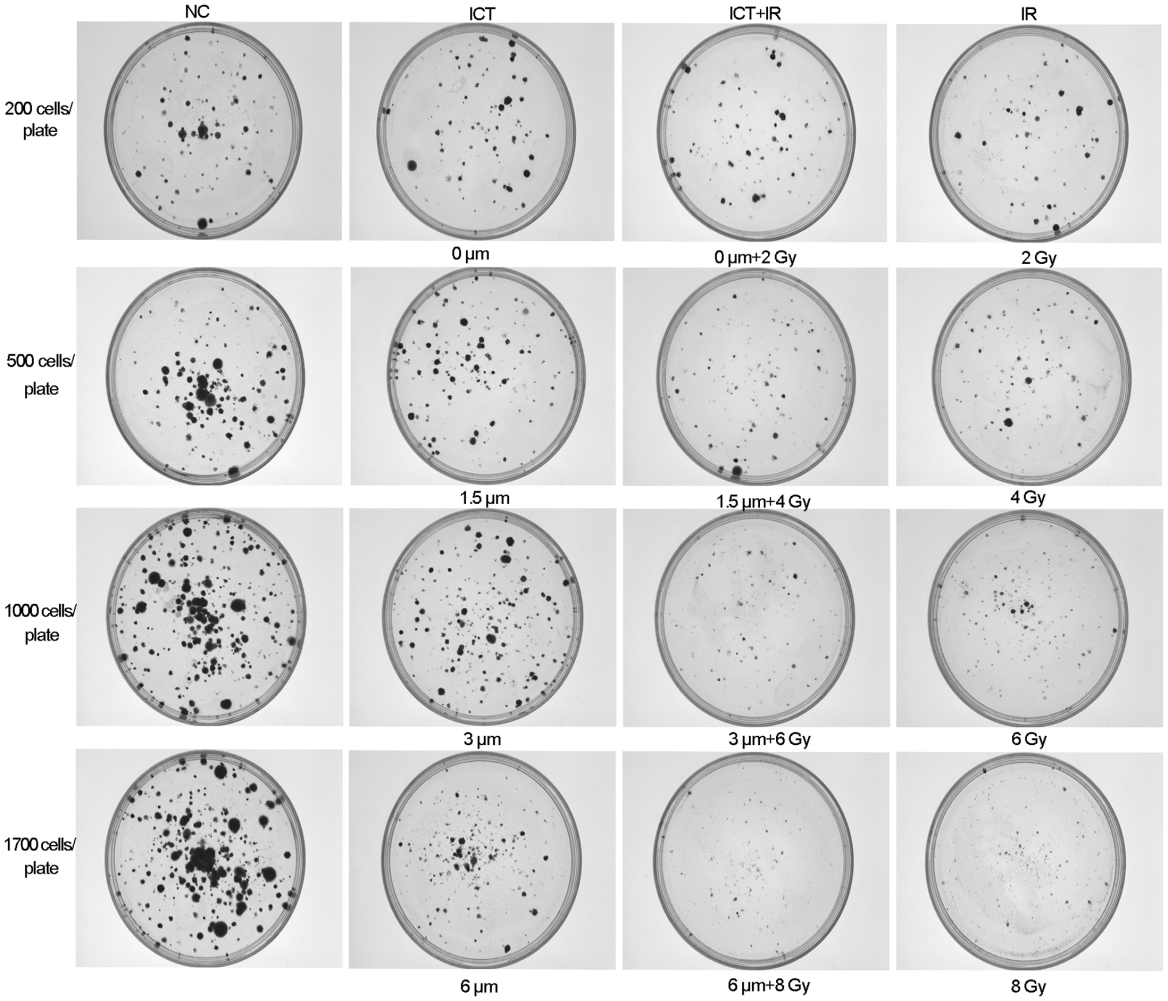
ICT acts synergistically with IR in clonogenic assay. The 4T1 breast cancer cells were treated with vehicle alone, ICT alone (0, 1.5, 3, or 6 µmol/L), radiation alone (0, 2, 4, 6 or 8 Gy) or combination of ICT with radiation. The cells were plated in 60 mm dishes at different densities, based on the stringency of the treatments. After 14 days, the colonies were stained with crystal violet, and the numbers of colonies (containing ≥50 cells) were counted using Image-Pro® Plus. The images represent the synergistic killing effects of ICT and IR on 4T1 cells.

The goal of this study is to explore if ICT can be used as a radiosensitizer to radiation, one of the mainstream treatment of various types of cancer. For this, the clonogenic assay was used to determine the extent of reproductive death caused by single treatment of ICT or radiation alone or both in combinations. Since the mouse 4T1 breast cancer cell line is one of the aggressive tumor cell lines that can grow as primary or metastatic tumor in BALB/c mice [26] and is relatively resistant to chemotherapy and radiotherapy [27], it was chosen as the targeted cells. The result (Table 1) demonstrated that when ICT was added to these highly malignant cancer cells, the IR dose required for reducing the fraction of colonies to 37% was dropped from 5.5 Gy to 4.7 Gy (at ICT 3 µM) or 3.7 Gy (at ICT 6 µM), respectively. It was deduced at the clinically relevant dose of 2 Gy, the ICT could reduce the survival fraction from 87% to 83% (at 3 µM) or 62% (at 6 µM), respectively. The treatment enhancement ratio (ER) increased to 1.18 (at 3 µM) or 1.28 (at 6 µM). The combination index (CI) was 0.38 or 0.19 and the dose reducing index (DRI) was 2.51 or 5.07 at ICT 3 µM or 6 µM, respectively. All data (Table 1 and Fig. 2) strongly suggests that ICT could exert a synergistic effect with radiation on aggressive cancer cells. Notably, the concentration of ICT needed for this sensitization effect was relatively low, which might be achievable *in vivo.*

**Table 1:** Icaritin enhances the radiosensitivity of murine 4T1 breast cancer cells.

Icaritin	D0 (Gy)	SF2	ER	CI*	DRI*
0 µM	5.5	0.87			
1.5 µM	4.8	0.85	1.30	4.95	0.20
3 µM	4.7	0.83	1.18	0.39	2.51
6 µM	3.7	0.62	1.28	0.19	5.07

D_0_, incremental dose required for reducing the fraction of colonies to 37%, indicative of single-event killing; SF2, surviving fraction of exponentially growing cells when irradiated at the clinically relevant dose of 2 Gy; ER, enhancement ratio; CI, combination index; DRI, dose reducing index.

* CI <1 and DRI >1 indicate a synergistic cytotoxic effect of icaritin and radiation.

### ICT Inhibits p-ERK1/2 and p-AKT Signaling

**Figure 3:**
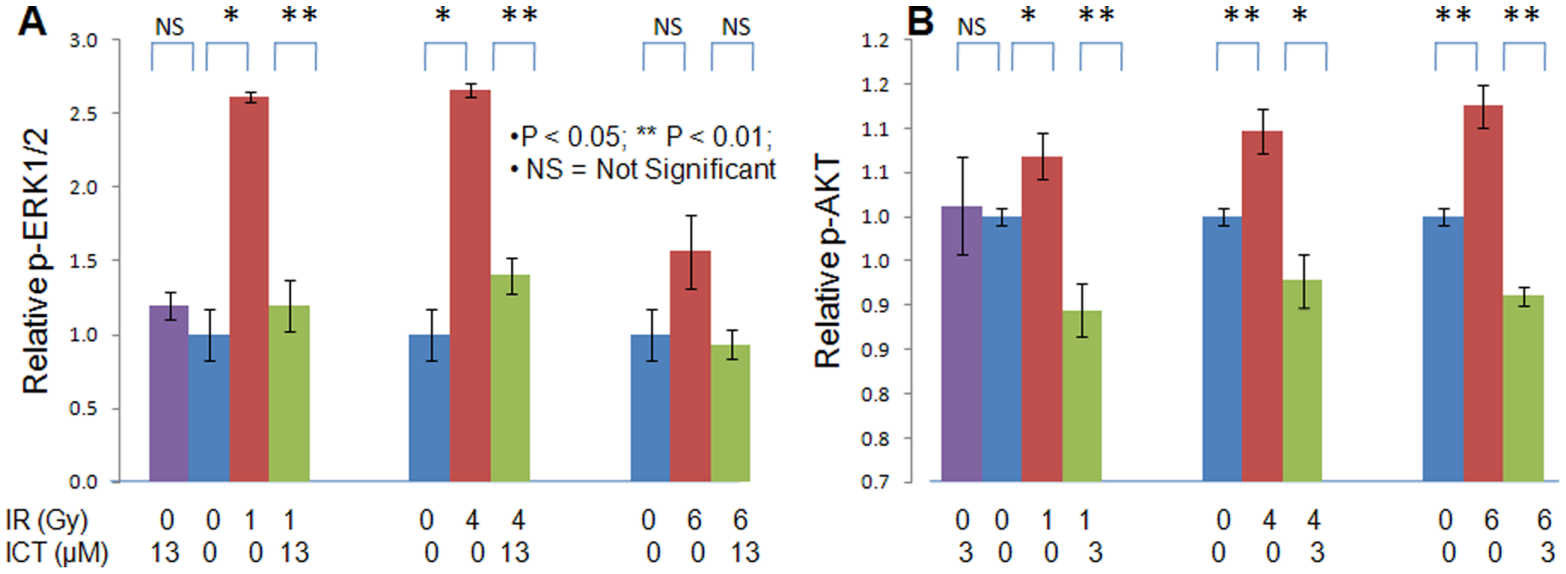
Icaritin inhibits irradiation-induced phosphorylation of ERK1/2 and AKT. (**A**) p-ERK1/2 assay. 4T1 cells were treated with vehicle alone or Icaritin at13 uM for 4 hr, and then irradiated with 0, 1, 4, or 6 Gy. Ten minutes later, the cells were fixed with 4% formalin for 5 min and subjected to an in-plate ELISA-like test with rabbit anti-phosphorylated ERK followed by horseradish peroxidase-anti-rabbit and a TMB substrate and A_450_ reading. (**B**) **p-AKT assay:** 4T1 cells were treated with vehicle alone or Icaritin at 3 uM for 24 hr, and then irradiated with 0, 1, 4, or 6 Gy; One hour later, the cells were fixed with 4% formalin and subjected to an in-plate ELISA-like assay using rabbit anti-phosphorylated AKT as first antibody followed by the same method as detection for phosphorylated ERK1/2. ∗ represents *p*<0.05; ∗∗ represents *p*<0.01; NS means not significant.

It is reported that radiation could trigger the activation of multiple signaling pathways, in particular, ERK1/2 and AKT [28], to promote some cancer cells growth after the stress of IR. To explore if ICT could counteract the undesirable survival signaling that helps cancer cells to escape the death and cancel-out the IR killing effect, the effect of ICT on IR-induced activation of p-ERK1/2 and p-AKT was studied with cell-based ELISA-like assay [23], [24]. Since two signal paths have its own sensitivity to ICT and its own phosphorylation timing upon IR stress, the assays were performed differently. For phospho-ERK1/2 assay, the 4T1 cells were treated with ICT for 4 hr and harvested 10 min after IR, while for phospho-AKT assay, the 4T1 cells were treated with ICT for 24 hr and harvested 1 h after IR. Figure 3 demonstrated that the IR at 1, 4, 6 Gy was able to trigger the phosphorylation of ERK1/2 and AKT, which could all be inhibited by ICT to their basal levels. It is clear that via suppressing IR-induced “rescue/survival” signaling, ICT acts together with IR to enhance the reproductive killing effect as demonstrated in the clonogenic assay (Table 1 and Fig. 2).

### ICT Enhances the Accumulation of G2-M Phase Upon IR

**Figure 4:**
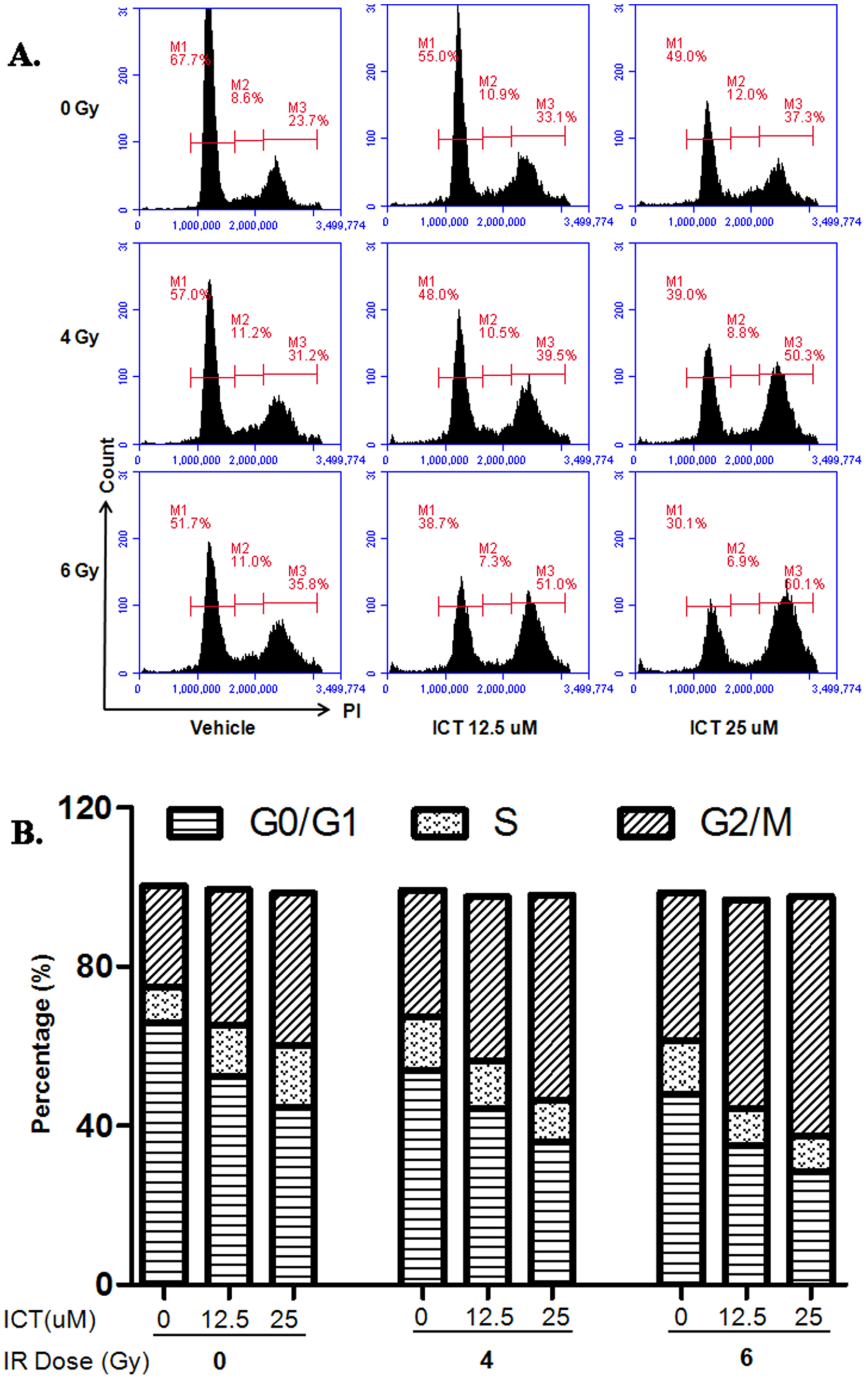
Accumulation of G2-M cell population after combination of ICT and IR. 4T1 cells were treated with ICT (0, 12.5, 25 µM), IR (0, 4, or 6 Gy), or ICT combined with IR. 72 hours after treatment, the cells were harvested and subjected to flow cytometry. (**A**) FCM images represent the fraction of cells in G0G1 phase (M1), S phase (M2) and G2-M phase (M3) after different single or combined treatment. (**B**) Bar graphs represent the percentage of cells in three different phases.

The unique feature of IR is to break DNA of cancer cells, which triggers the activation of P53, a blocker on G_2_/M phase and induces the apoptosis [29]. To determine if ICT could enhance this G2/M blockage, the 4T1 cells were treated with different doses of ICT (0, 12.5 and 25 µM) or IR (0, 4 or 6 Gy), or ICT combined with IR for 72 hours, and then the fractions of cells in different cycles were assessed with flow cytometry. Results (Fig. 4) showed that while IR (4 or 6 Gy) alone could shift the G_2_/M (indicated as M3) cells from 23.7% to 31.2% or 35.8%, the combination of ICT at 12.5 µM made this shift up to 39.5% or 51.0%, and at 25 µM to 50.3% or 60.1%, respectively. While ICT or IR alone could slightly increase fraction of cells in S phase (indicated as M2), the combination of both reduced the cells in S phase. With the increased fraction of G_2_/M phase, the G_0_G_1_ phase cells reduced (indicated as M1, Fig. 4 A). The cells arrested at G_2_/M phase would be pushed into apoptosis [30].

### Combination of ICT and IR Increases Cell Apoptosis

**Figure 5:**
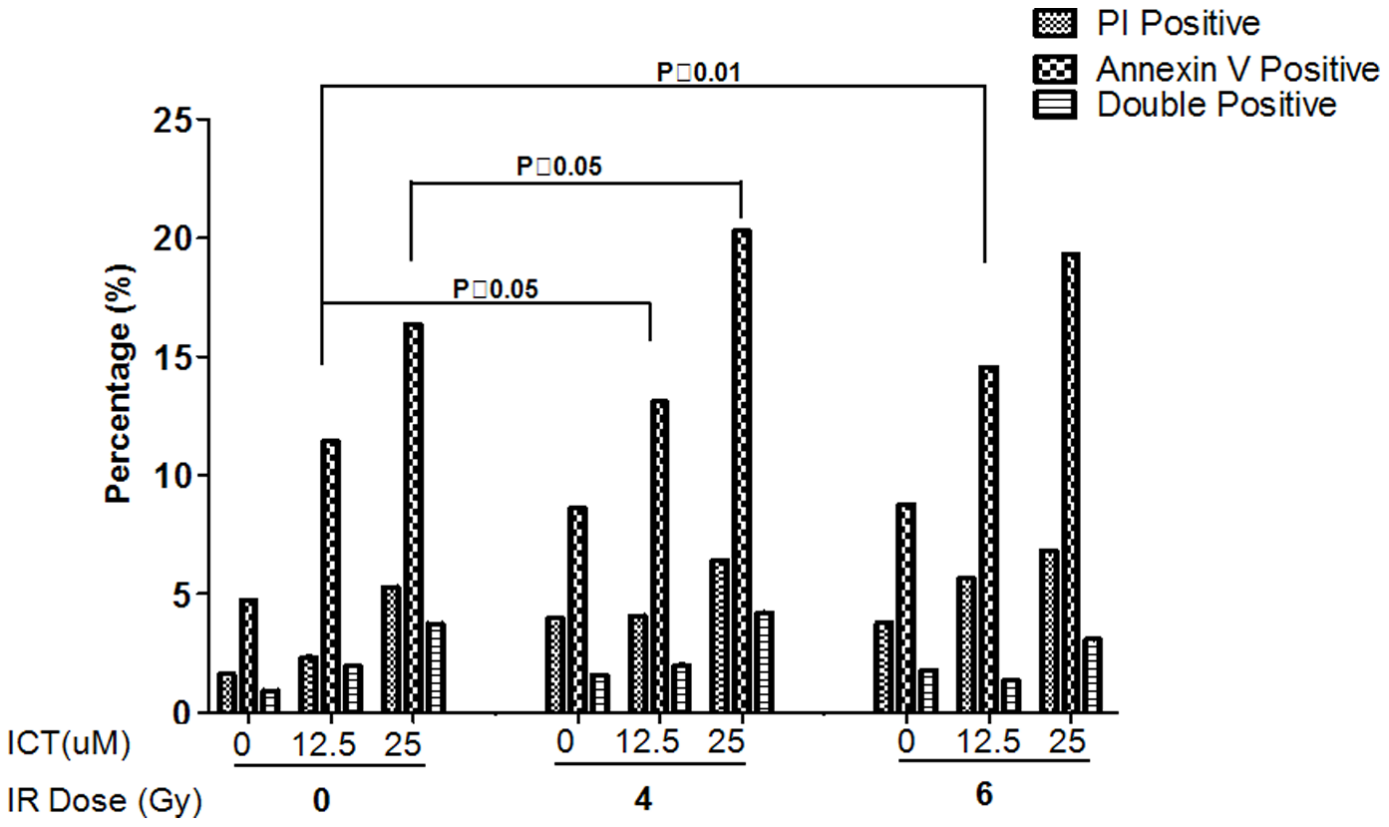
Combination of ICT and Radiation Increased Cell Apoptosis. 4T1 cells were treated with ICT (0, 12.5, 25 µM), IR (0, 4, or 6 Gy), or ICT combined with IR. 72 hours after treatment, the cells were harvested and stained with Annexin V and PI. Combination of ICT and IR enhanced apoptosis.

The cells blocked in G2-M phase unable to pass into G_0_G_1_ phase are likely to take the path of apoptosis. To determine if this is the case, the 4T1 cells were treated with ICT (at 0, 12.5 or 25 µM), or radiation at (0, 4 or 6 Gy), or ICT combined with radiation. 72 hours after treatment, the cells were harvested and stained with Annexin V and PI for three subpopulations: apoptotic cells (Annexin V positive), early dead cells (Annexin V and PI double positive) and dead cells (PI positive). The results (Fig. 5) showed that the while single treatment could increase three subsets of sick cells, the combined treatment could promote more cancer cells into these three subsets, especially when IR 4 or 6 Gy combined with ICT 12.5 µM or 4 Gy IR with 25 µM ICT (P<0.05). ICT with IR indeed acts together to enhance the killing effect on cancer cells.

### ICT Inhibits Angiogenesis in the Chick Embryo Chorioallantoic Membrane (CAM)

**Figure 6:**
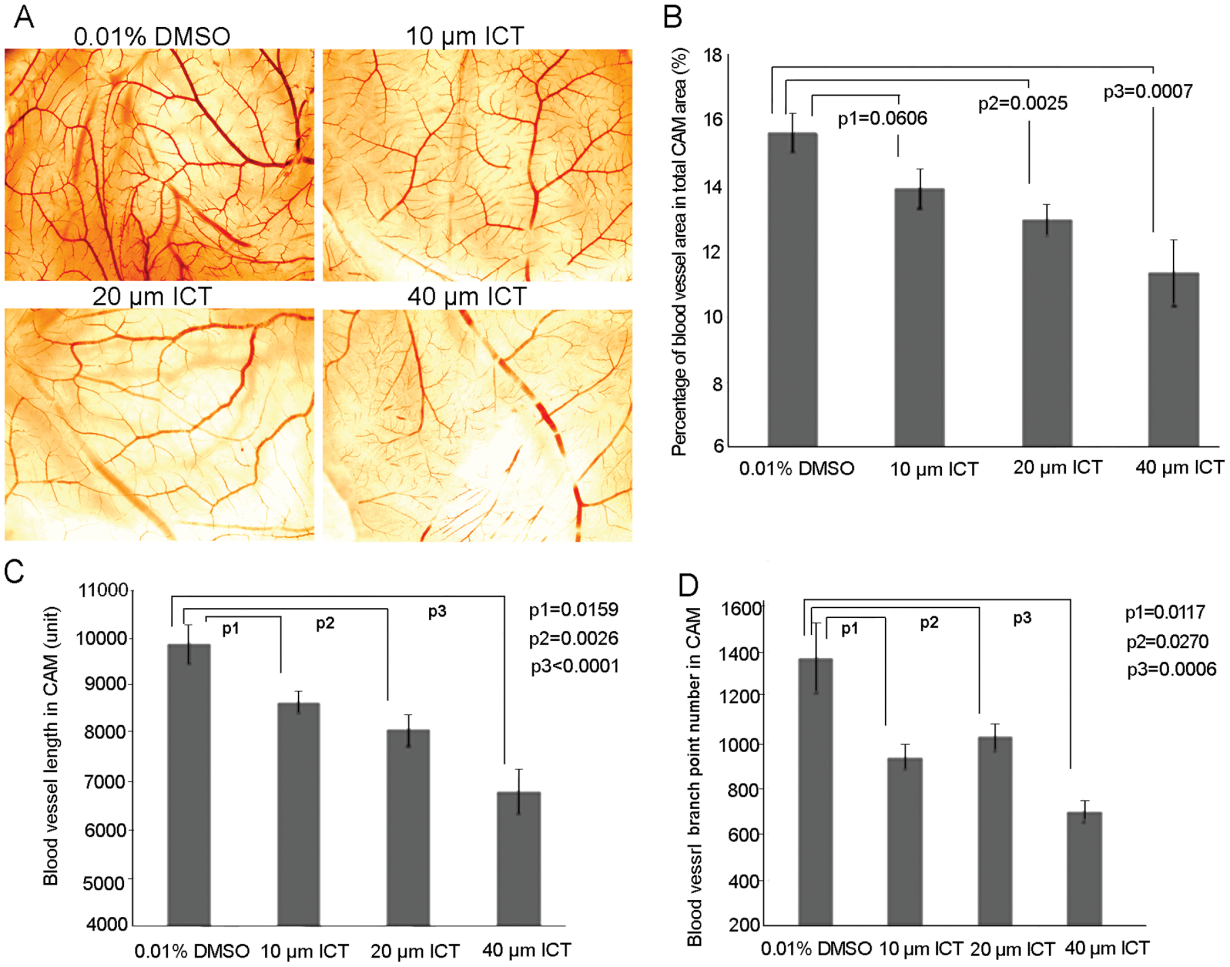
Icaritin inhibits angiogenesis in the chick embryo chorioallantoic membrane (CAM) assay. The fertilized eggs were incubated at 37°C for eight days and then divided into groups (8 eggs/group). 100 µl of 0, 10, 20 or 40 µM ICT was added to the top of CAM. After incubation for 3 more days, the CAM of each alive egg was harvested and placed individually in 6 well plate. (A) Images of the blood vessels of each CAM after treatment with various concentrations of ICT for 8–10 days were captured using Motic Images Plus®. (B) Mean vessel area as a percentage of the total area, (C) mean vessel length and (D) mean number of branch points were obtained by using Image Pro® analysis software.

To kill the tumor, besides to knock-down their reproductive ability, the blocking of their blood supply is also crucial. To determine if ICT could not only enhance the IR effect, but also suppress the angiogenesis, CAM assay was performed in 8 day fertilized eggs. Figure 6A showed that the newly-formed vessels were reduced in an ICT dose-dependent manner. Quantitative analysis of properties of angiogenesis demonstrated that ICT significantly decreased the vessel area (Fig. 4B), vessel length (Fig. 4C) and number of dendrites (Fig. 4D) in a dose-dependent manner, suggesting that ICT possesses the capacity for anti- angiogenesis, which is likely to help the tumor shrinkage *in vivo*.

## Discussion

This study is to explore the synergistic effect of ICT with radiation. For the first time, we demonstrate that ICT enhances the radiosensitivity of the highly malignant 4T1 breast cancer cells. ICT is capable to reduce IR dose that needed to kill cancer cells (D_0_ reduced) and allow 2 Gy IR to kill more cancer cells (SF_2_ reduced). Two key criteria for synergistic effect are met: combination index (CI) <1 and dow reducing index (DRI) >1. All these data are obtained from the gold standard clonogenic assay, the best evidences for reproductive death induced by IR [31].

The action mechanisms by which the synergistic effect of ICT with radiation exerted are associated with several aspects as indicated by our data.

First, IR is an overwhelming physic insult to cells. To counteract the IR stress, some cancer cells instantly activate their own survival signaling, such as ERK1/2 and AKT paths, which might accelerate the repopulation and compensate the lost cells. The ICT could block the activation (phosphorylation) of ERK1/2 and AKT and disrupt the protection process (Fig. 3). Therefore, more reproductive death is observed (Table 1 and Fig. 2).

Secondly, it is well-known that IR could activate P53 and push cell staying G_2_/M phase then subject to internally triggered apoptosis. In addition, the cells in G_2_/M phase are most sensitive to IR killing. ICT could enhance the cell accumulation in G_2_/M phase (Fig. 4 A and B). The more cells abnormally are blocked-in or accumulate in the G_2_/M phase, the more cells could die due to the internal apoptosis or external IR killing. The molecules involved in this process might include these in the P53, ERK1/2 and AKT paths. This might be the major reason that ICT can be a radiosensitizer.

Thirdly, IR causes cell death mainly via necrosis and apoptosis. When IR dose is high enough, it strongly disrupts the cellular macromolecules, including structure protein, functional lipids, enzymes and DNA, leading directly to necrosis. However, if the IR dose is low and cell has the capacity to repair the damage, it can have two scenarios: (1) fidelity repair as all repaired molecules carrying out the function normally, in which cells are fully recovered; and (2) infidelity repair as some of molecules mutated with abnormal functions, which could trigger the apoptosis to clean-up these mutated cells to ensure that the repopulating cells are normal and healthy. Our data (Fig. 5) shows that ICT could also induce both necrosis and apoptosis in a dose-dependent manner. Since the induction of necrosis and apoptosis by IR and ICT utilizes two different mechanisms, a physic and chemic insult, respectively, the combined use of IR and ICT would increasingly damage the cancer cells through these two different paths, which makes the insulted cancer cells even more difficult to repair damages from each insult, leading to a synergistic killing effect on the treated cells as showed in Table 1 and Fig. 2.

There are several studies exploring the action mechanism of ICT. Some of them are consistent with our data. For example, ICT treated human MCF-7 and MDA-MB-453 breast cancer cells could also induce cancer cell arrested in G2/M phase and cell death [21], similar to what we found in mouse 4T1 breast cancer cells. Other molecules are also involved in ICT action, such as upregulating retinoblastoma protein (Rb), p27^Kip1^ and p16^Ink4a^ protein expression, downregulating phosphorylated retinoblastoma protein (pRb), Cyclin D1 and CDK4 protein expression, reducing mitochondrial transmembrane potential [11], [12] and suppressing JNK activation [14].

Clinical studies have demonstrated the benefits of adjuvant radiotherapy for improving disease-free survival, overall survival and local control, and reducing disease recurrence after surgical resection in breast cancer patients [17]. However, radiotherapy is associated with side effects, including epidermitis [25], lung fibrosis [32] and an increased risk of cardiovascular disease [33]. The discovery of novel agents which sensitize malignant cells to radiation would increase tumor response, while minimizing toxicity to the surrounding organs by lowering the effective therapeutic dose. ICT at a concentration of 3 µM to 6 µM enhanced the radiosensitivity of 4T1 cells and potentially lowering the IR dose to achieve the same killing effect at higher IR dose, and thereby reducing the IR side effect.

Another major finding of this study is that ICT significantly inhibited angiogenesis in the CAM assay. Since the angiogenesis plays an important role in the growth and progression of solid tumors [34], the anti-angiogenesis effect would further enhance the ICT anti-tumor effect by its own or combination with IR *in vivo*.

In summary, this study demonstrates that ICT synergistically acts with IR to enhance the reproductive death of highly malignant 4T1 breast cancer cells via suppressing IR-induced activation of ERK1/2 and AKT survival paths, enhancing the cancer cell G_2_/M arrest and apoptosis. These results suggest that pharmacological approaches using ICT as a new radiosensitizer in combination with IR could increase the effectiveness of IR cancer treatment, which could have important implications for the development of novel radiotherapy strategies.
